# MAX: a simple, affordable, and rapid tissue clearing reagent for 3D imaging of wide variety of biological specimens

**DOI:** 10.1038/s41598-022-23376-6

**Published:** 2022-11-14

**Authors:** Boram Lee, Ju-Hyun Lee, Dai Hyun Kim, Eun Sil Kim, Bo Kyoung Seo, Im Joo Rhyu, Woong Sun

**Affiliations:** 1 Department of Anatomy, Brain Korea 21 Plus Program for Biomedical Science, Korea University College of Medicine, Seoul, 02841 Republic of Korea; 2 Department of Dermatology, Korea University College of Medicine, Seoul, 02841 Republic of Korea; 3 Department of Radiology, Korea University Ansan Hospital, Korea University College of Medicine, Ansan, 15355 Republic of Korea

**Keywords:** Fluorescence imaging, Immunological techniques, Optical imaging

## Abstract

Transparency of biological specimens is crucial to obtaining detailed 3-dimensional images and understanding the structure and function of biological specimens. This transparency or tissue clearing can be achieved by adjusting the refractive index (RI) with embedding media and removing light barriers such as lipids, inorganic deposits, and pigments. Many currently available protocols consist of multiple steps to achieve sufficient transparency, making the process complex and time-consuming. Thus, in this study, we tailored the recipe for RI adjustment media named MAX based on the recently reported MACS protocol to achieve a single-step procedure, especially for ECM-rich tissues. This was achieved by the improvement of the tissue penetrability of the RI-matching reagent by combining MXDA with sucrose or iodixanol. While this was sufficient for the 3D imaging in many applications, MAX can also be combined with modular processes for de-lipidation, de-coloration, and de-calcification to further maximize the transparency depending on the special features of the tissues. Our approach provides an easy alternative for tissue clearing and 3D imaging.

## Introduction

Tissue-clearing techniques have long been recognized as useful tools for exploring the 3-dimensional (3D) architecture of biological specimens at high cellular resolution, in the absence of a physical section^1–4^. Although these techniques have been primarily developed for the clearing of mammalian soft organs such as the brain^2,3^, successive tailoring of the procedure, suitable for a wide variety of biological specimens from plants^5,6^, marine invertebrates^7,8^, insects^8–10^, vertebrates^1,3,11–13^, and humans^14^, have broadly impacted biological sciences. Each biological specimen is composed of different macromolecules that have distinct optical properties; the need for the tailoring protocols for each specimen has caused an exponential increase in tissue-clearing protocols^16–18^. This has resulted in difficulty in choosing a suitable protocol for a given specimen and a need to establish a simplified workflow universally applicable to a variety of biological specimens has arisen.

Tissue clearing is achieved by correcting the optical properties of the biological specimen via refractive index (RI) matching, de-lipidation, and in some cases, de-coloration^1,2,13,19–23^. The efficiency of these processes is strongly associated with hydrophobicity/hydrophilicity of biological specimens. For instance, tissue transparency is differentially affected by lipid content and the average RI value^17,24–27^. In lipid-rich organs, de-lipidation is essential to achieve a substantial degree of transparency. However, de-lipidation has little effect on the transparency of tissues with high protein content owing to the rich extracellular matrix (ECM) e.g., tendons, skin, and bones. Thus, RI adjustment contributes more to achieving transparency in these tissues, and the use of materials exhibiting high RI and good tissue penetrability is desirable.

Recently, significant efforts have been made to identify chemicals for tissue clearing^28–31^. In an attempt to search of suitable RI reagents, MXDA was identified as a new tissue clearing chemical with high RI in aqueous solution^28^. MXDA has very high RI index (RI = 1.57), with low viscosity and relatively affordable cost, raising this chemical as a good alternative of other RI adjustment media. One limitation of MXDA was that the high concentration of MXDA cause the dehydration of the tissue, impeding the diffusion of the MXDA into the deep tissue inside, making the tissue clearing reaction difficult. Thus, Zu et al*.* proposed that stepwise incubation of biological specimens with increasing concentrations of MXDA can be used for whole-organ or organism clearing^32^. In our study, we improved the RI solution based on MXDA to achieve a better tissue penetrability without reduction of the tissue clearing activity, allowing a single-step tissue-clearing protocol. We named this MAX (MXDA-based aqueous RI adjustment solution X). Furthermore, MAX can be used as a vasatile tool to clear a wide variety of biological specimens with modular inclusion of other steps for de-lipidation, de-coloration, and de-calcification.

## Results

### Optimization of MAX reagents with rat tail tendon (RTT) samples

**Figure 1:**
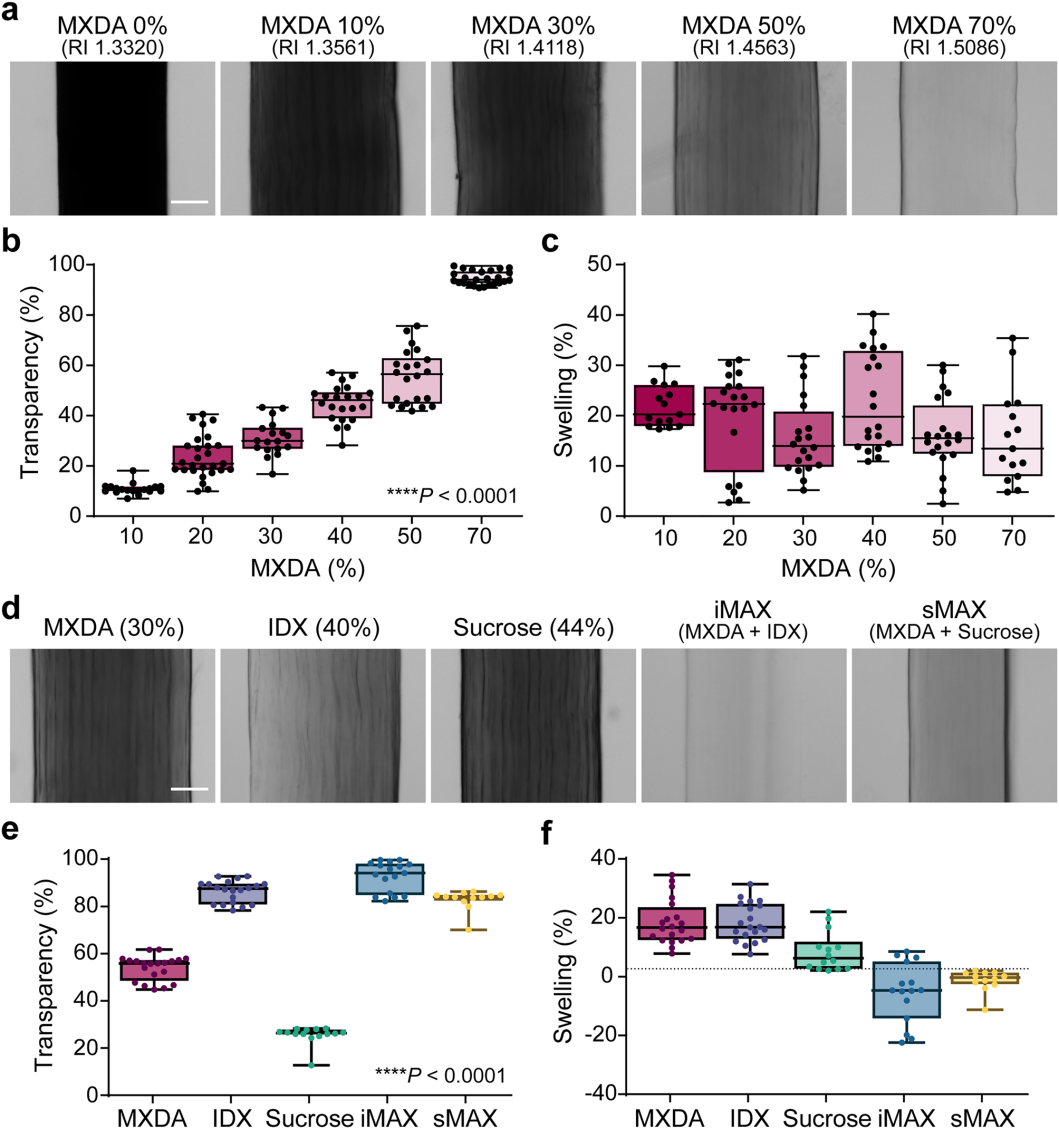
Optimization of the MXDA-based extracellular matrix (ECM) clearing media. (**a**) Changes in the size and transparency of the rat tail tendon (RTT) depending on the concentration of the MXDA. Right graphs show the densitometric quantification results. Scale bar, 100 μm. (**b**) Quantification of RTT transparency and (**c**) swelling percent in different MXDA concentrations N = 9 animals. Data spots were obtained from 1–3 different RTTs from individual animals. (**d**) Effects of MXDA (40%) with iodixanol (IDX) or sucrose on the transparency and size of RTT. Both mixtures (iMAX or sMAX) were adjusted to RI = 1.47. Scale bar, 100 μm. (**e**,**f**) Quantification of transparency (**e**) and swelling percent (**f**) in the mixtures, N = 6 animals. Data spots were obtained from 1–3 different RTTs from individual animals. Standard one-way ANOVA with Tukey’s multiple comparison test was used for multiple comparisons (**b**,**e**).

To test the effect of MXDA on dense ECM tissues, we chose the RTT and quantified the concentration-dependent changes in transparency and tissue size (Fig. 1a). The RTT transparency increased owing to increased RI of the solution with increasing MXDA concentration in the solution (Fig. 1b). MXDA also induced tissue swelling (Fig. 1c). The hyper-hydration effect of MXDA was previously reported and speculated to be associated with the chaotropic properties of MXDA, which causes protein denaturation^28^. These features intensify the tissue transparency by diluting biomolecules and enhancing the media penetrability. In contrast, a high concentration of MXDA caused RTT dehydration and hardened (Supplementary Fig. 1a). Urea, which shares significant structural similarity with MXDA, also exhibits a bi-phasic dehydration effect when a high concentrations of urea is used. It inadvertently increases osmolality of the media and promotes water removal from tissues/proteins^17,33^. Dehydration removes water, which has low RI; therefore, dehydration can increase the homogeneity of tissue components and reduce light scattering. Further, we observed that the edge of the tissue, i.e., the interface of the tissue and media, exhibited darker shades at higher MXDA concentrations. We speculated that this was associated with the formation of impeding interface zone between a high concentration of MXDA in the RI media and water in the specimen. To obviate these potentially negative effects (i.e. tissue shrinkage, dehydration, and impeding interface formation) of high concentration of MXDA, we decided to determine the combination of MXDA and iodixanol or sucrose, which also possesses a suitable RI with normal osmolarity^29^. We measured the RI of the mixture with step concentrations and finally determined the mix of MXDA (30%): iodixanol (40%) and MXDA (30%): sucrose (45%) (sucrose RI is approximately 1.47, which is the known average RI value of the biological samples). We named this iodixanol: MXDA mix iMAX, and sucrose: MXDA mix sMAX in the following experiments. Mixtures of MXDA and iodixanol or sucrose additively increased transparency without the tissue swelling effect (Fig. 1d–f). A similar additive effect on tissue transparency was observed in mouse skin specimens (Supplementary Fig. 1b).

### Tissue/organ clearing with or without de-lipidation

**Figure 2:**
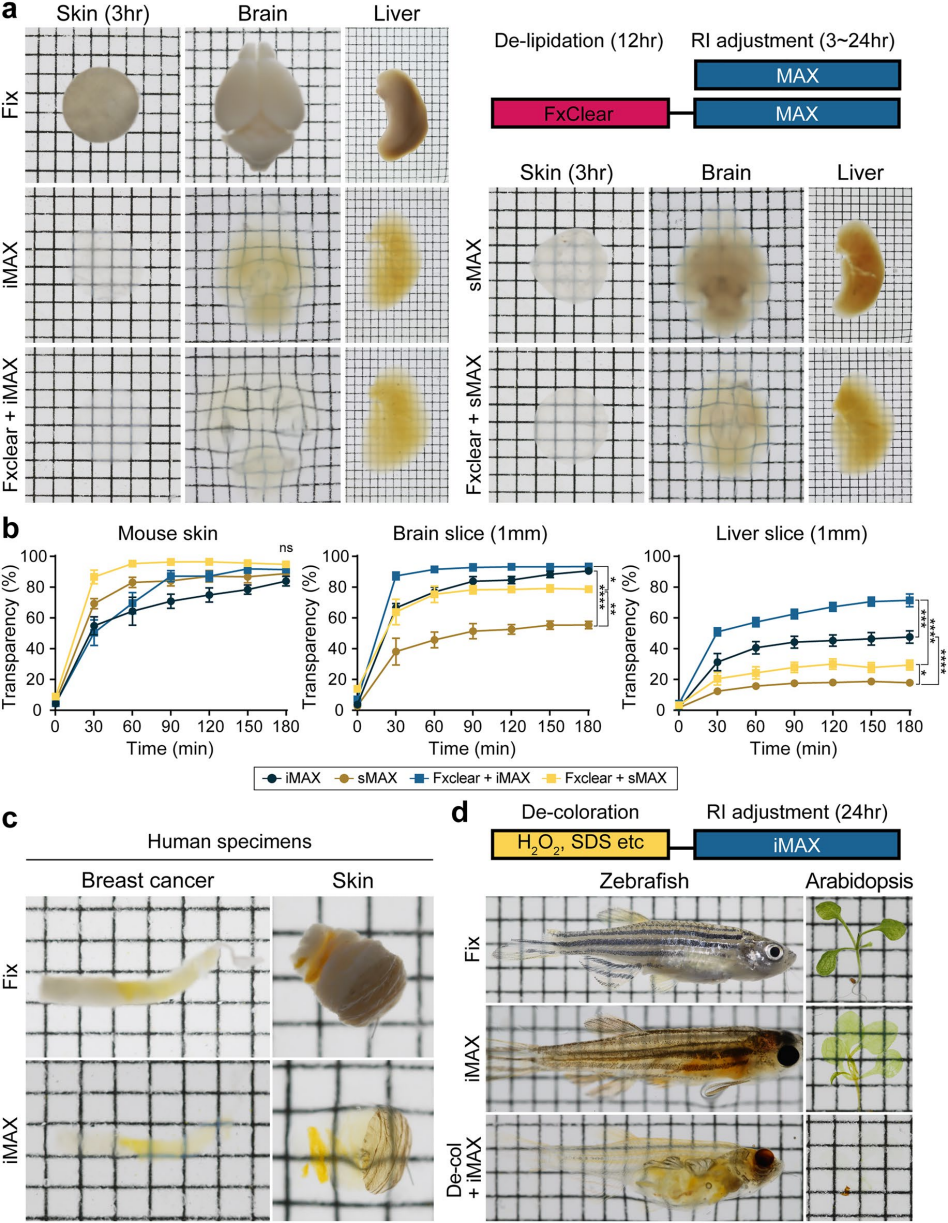
Organ clearing in MAX solutions. (**a**) Fixed mouse organs are immersed in the iMAX or sMAX solutions 3 h (skin) or overnight (brain and liver) with or without FxClear de-lipidation steps. Specimens are placed on the 2-mm grid paper. (**b**) Kinetics of specimen clearing in the MAX solutions. Specimen are prepared with or without FxClear step, N = 4 animals. The unpaired two-tailed *t*-test was used to analyze the differences in maximum transparency (180 min) between the groups (ns, not significant; *p < .05; **p < .01; ***p < .001; ****p < .0001). (**c**) Clearing of human specimen in iMAX solution. (**d**) Clearing of whole adult zebrafish and Arabidopsis in iMAX media. Specimen are de-colorized by treatments with H_2_O_2_ (zebrafish) or sodium dodecyl sulfate (SDS) (Arabidopsis). All specimens are placed on the 2-mm grid paper. Images were acquired using a Digital Single Lens Reflex camera (Canon).

Although we optimized MAX as a single-step clearing reagent for connective tissues, we tested the clearing efficiency of MAX for many whole mouse organs, including the lungs, muscle, brain, kidney, spleen, and liver (Fig. 2a and Supplementary Fig. 2). Considering that MAX does not contain reagents for de-lipidation, we also compared MAX with or without pretreatment of electrophoretic tissue clearing by FxClear^34^. In the iMAX solution, most of the organs reached a comparable value of transparency, even in the absence of FxClear pretreatment, suggesting that the de-lipidation step only partially contribute to the efficiency of tissue clearing, and iMAX is sufficient for mouse whole-organ clearing. sMAX appeared to be less efficient, and de-lipidation significantly improved the organ transparency. To precisely determine the efficiency and duration for organ clearing, we selected three organs (skin, 1-mm sliced brain, and 1-mm sliced liver) and examined the profile of organ transparency for 3 h (Fig. 2b and Supplementary Movie S1). There were significant differences in the maximal transparency and clearing kinetics achieved by both iMAX and sMAX depending on the organs and the addition of FxClear de-lipidation process exhibited beneficial effects on the transparency of the brain and liver slices whose transparencies were not sufficient by MAX alone. Interestingly, although the iMAX-based tissue clearing was relatively efficient in most organs tested, sMAX clearing was more rapid than iMAX for the skin clearing (iMAX vs. sMAX, P values < 0.0001; FxClear + iMAX vs. FxClear + sMAX, P < 0.0001; two-way ANOVA test with Bonferroni correction). This was primarily owing to the even clearing of the periphery and center regions of the tissues in sMAX media. Thus, when quantified the clearing kinetics in the periphery and center regions separately, the difference of iMAX and sMAX at the periphery was minimized, while that at the center became more striking (Supplementary Fig. 3a; iMAX vs. sMAX, P < 0.0001; FxClear + iMAX vs. FxClear + sMAX, P < 0.0001; two-way ANOVA test with Bonferroni correction). These results suggest that iMAX and sMAX have different properties of tissue clearing, and the empirical check with target specimen may be required.

Next, we compared the MAX reagent with other chemicals offering single-step tissue clearing. We chose two reagents, CUBIC-mount (CM) which is derived from CUBIC clearing protocol, and RIMS which is commercially available (Supplementary Fig. 3b and c)^2,13^. On RTT clearing, RIMS was equivalent to the iMAX reagents, while CM was significantly inferior. On the other hand, RIMS showed poor clearing kinetics on skin clearing comparing to the sMAX (iMAX vs. RIMS, P = 0.0119; sMAX vs. RIMS, P = 0.0003; two-way ANOVA test with Bonferroni correction). Considering that MAX reagent is much cheaper than RIMS, MAX can be an excellent alternative of RIMS.

When biopsy samples from human patients were tested with iMAX, both skin and breast tumor samples were readily cleared (Fig. 2c), indicating that iMAX can potentially be used for histological analyses of biopsy samples. To test whether iMAX can be applied to other biological specimens, we chose two specimens with different features. Adult zebrafish was cleared by simple dipping into an iMAX solution for 24 h (Fig. 2d). However, the striped pattern of zebrafish remained intact as it is derived from melanin pigments and can be bleached by only low concentrations of H_2_O_2_. Pretreatment of zebrafish with H_2_O_2_ can efficiently remove the colored pigment and achieve better transparency^33,35^. While the second sample consisting of white mushrooms (*Agaricus bisporus*) were cleared by 24 h incubation with iMAX solution (Supplementary Fig. 4a), green plants (*Arabidopsis*) in this sample were treated additionally with sodium dodesyl sulfate (SDS; de-coloration step) to obtain whole plant transparency^36^. The iMAX procedure did not require a change in solution to achieve equivalent transparency, which can be achieved by the 3-step procedure using iMAX’s parental version, MACS^28^ (Supplementary Fig. 4b). Collectively, these results suggest that the iMAX protocol can be combined with other modules, if necessary, to achieve maximal transparency, depending on the biological specimen.

### Label-free imaging of MAX-cleared brain

**Figure 3:**
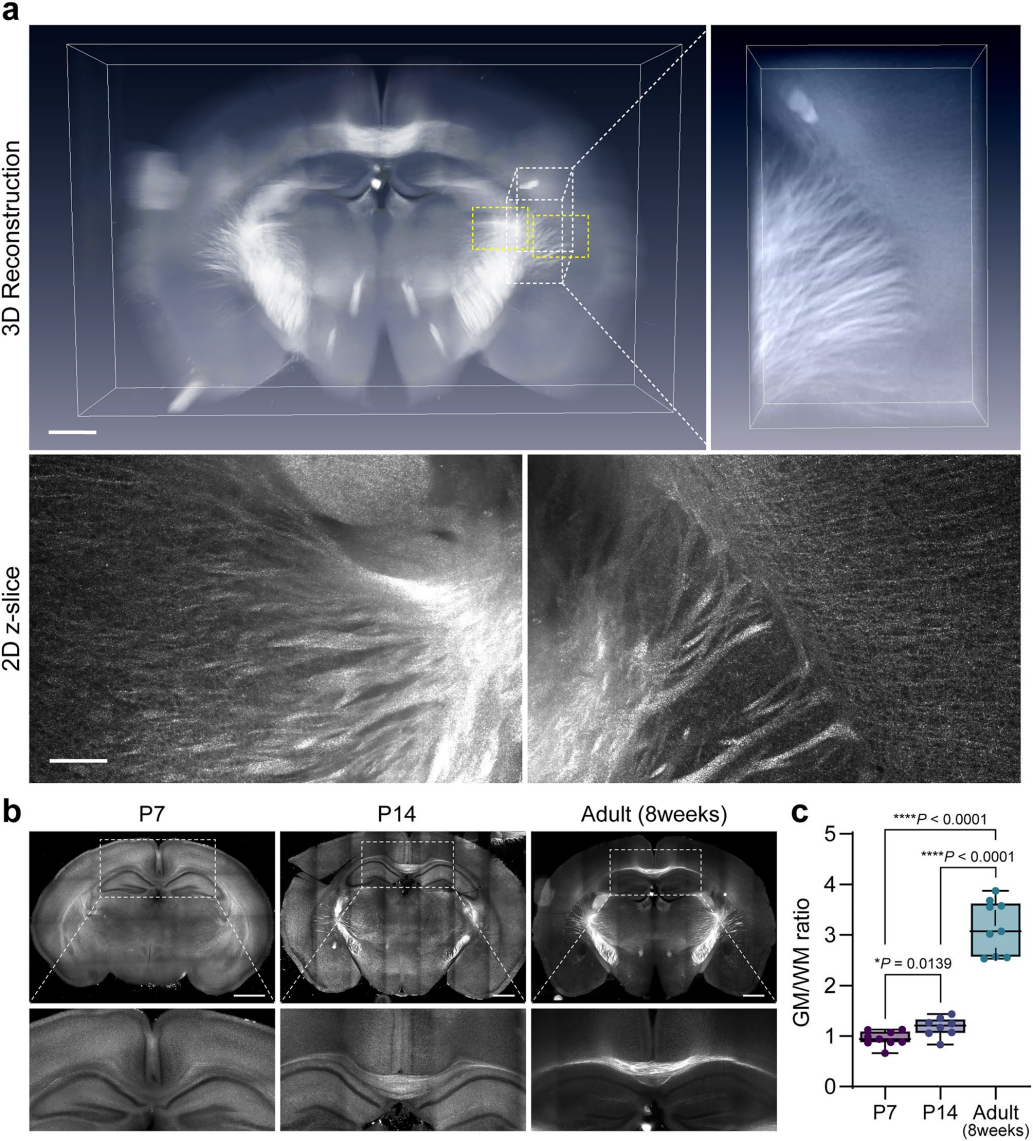
Label-free imaging of mouse brain in iMAX solution. (**a**) A 2-mm thick coronal slice of mouse brain is imaged with selective-plane illumination microscopy (SPIM) and processed with Amira software for 3D reconstruction. Boxed area with while dotted line is magnified and shown in the left panel. Single-plane images of the boxed areas with yellow dotted lines are shown at the bottom panels. Scale bar, 2 mm for upper panel and 200 μm for lower panel. (**b**) Development of mouse white matters during the 7 days, 14 days, and 8 weeks after birth as imaged by the label-free SPIM imaging. Lower panels are magnified images of corpus callosum region. Scale bars, 1 mm. (**c**) Quantification of grey matter/white matter (GM/WM) signal ratio. N = 8 each stage. T-test was used to analyze the difference between groups.

Previously, we demonstrated that label-free imaging with selective-plane illumination microscopy (SPIM) microscopy is applicable to whole-organ imaging in combination with a tissue-clearing procedure^24^. The source of label-free signals is multiple organic materials including lipids. Since iMAX preserves a large subset of lipids in the organ, we tested whether the label-free imaging technique is applicable for organ imaging (Fig. 3). As expected, nerve tracks were readily recognizable in the iMAX-processed adult mouse brain (Fig. 3a and Supplementary Movie S2). Details of individual fiber tracks radially projected in the cerebral cortex or thalamic regions were readily detectable in the high-resolution imaging in 2D slices as well. It is known that myelination primarily occurs during postnatal development^37^, and the corpus callosum becomes progressively noticeable during postnatal development (Fig. 3b and 3c). These results suggest that the maturation of nerves with dense myelination can be visualized using label-free methods.

### Fluorescence imaging of MAX-processed samples

**Figure 4:**
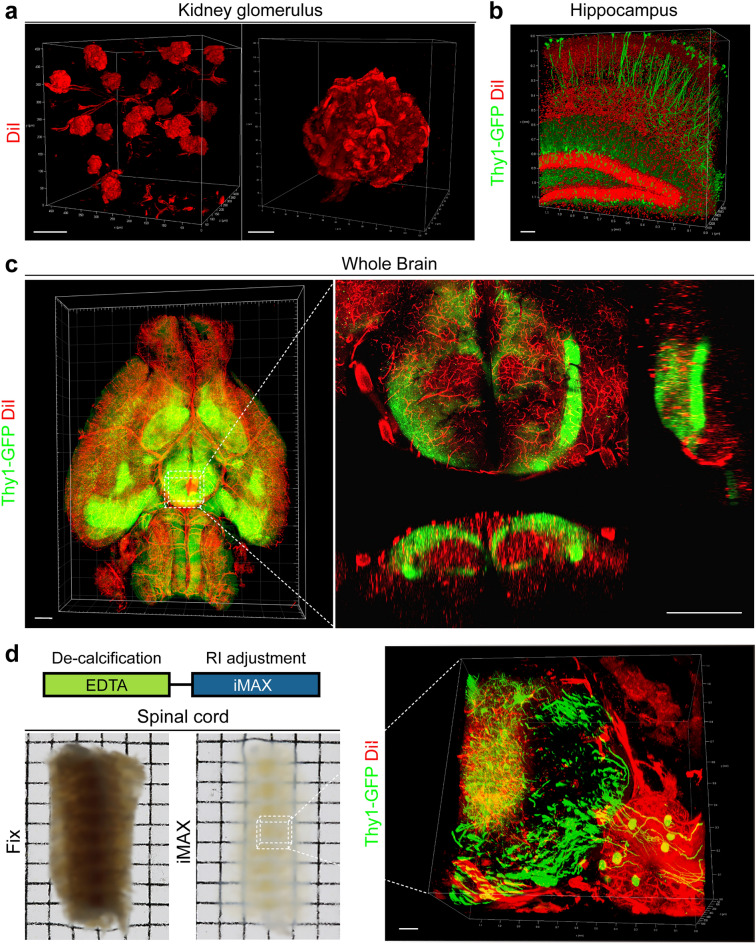
iMAX-based 3D imaging of the mouse organs. (**a**) Kidney glomeruli capillaries are visualized with CM-DiI infusion. Scale bar, 100 μm for left panel and 20 μm for right panel. (**b**) Green fluorescence protein (GFP) signals in the hippocampus of Thy1-GFP mice. Nuclei are counter-stained with PI. Scale bar, 100 μm. (**c**). Imaging of whole Thy1-GFP mouse brain. Red fluorescence was derived from the blood vessels pre-labeled by CM-DiI infusion. Right panel shows the orthogonal view of the ventral brain regions. Scale bars, 1 mm. (**d**) Imaging of spinal cord in the vertebrae. For the bone clearing, de-calcification was executed prior to the iMAX immersion. The specimen are placed on the 2-mm grid paper. Fluorescence signals from CM-DiI and Thy1-GFP are detected in the middle portion of the specimen as marked by black box. Scale bar, 100 μm. All fluorescent images were acquired using a TCS SP8 confocal laser-scanning microscope, and processed with Las X software (**a**,**b**,**d**) or Imaris (**c**) for 3D visualization.

Since the MAX processing does not include de-lipidation process, lipophilic dye labeling is preserved after the process. Thus, perfusion-based vascular labeling with CM-DiI was well conserved after iMAX processing, and the kidney’s glomerular structure was perfectly detected (Fig. 4a and Supplementary Movie S3). In addition, Green fluorescence protein (GFP) signals in the thy1-GFP mouse brain were readily detected, suggesting that iMAX can be used for fluorescence-based imaging. We were able to visualize the histoarchitecture and distribution of GFP-labeled neurons in the hippocampus by co-labeling the nuclei with propidium iodide (Fig. 4b). Transparency achieved by iMAX immersion was sufficient to visualize the entire mouse brain (Fig. 4c and Supplementary Movie S4). By including the de-calcification step of EDTA treatment, we also achieved GFP and CM-DiI imaging in the spinal cord enclosed in the vertebral bone (Fig. 4d). Using this procedure, bipolar axon projections of sensory neurons in the dorsal sensory ganglion were clearly observed.

### Immunofluorescence imaging of MAX-processed samples

**Figure 5:**
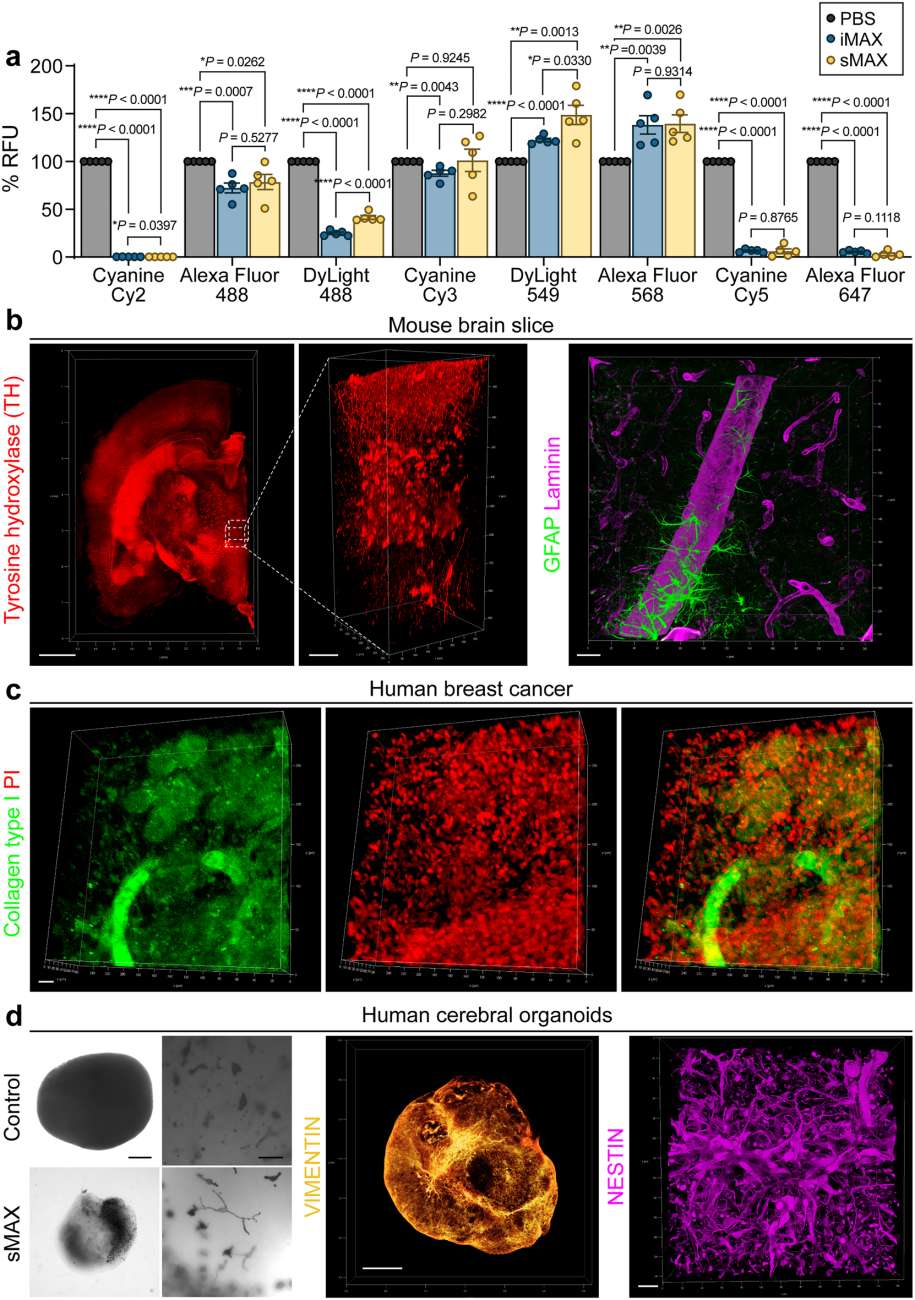
Immunofluorescence 3D imaging with sMAX media. (**a**) Quantification of fluorescence signal changes by the mix with MAX media. Fluorescence-labeled antibody was diluted 10 times with PBS, iMAX, and sMAX, and the fluorescence signal intensities were measured with fluorometer 18–24 h later. (**b**) Reconstituted 3D image of 2-mm brain slice. Samples is stained with tyrosine hydroxylase (TH), and then immersed in sMAX (**b**, left). Scale bars, 1 mm for whole image and 100 μm for ROIs. Visualization of blood vessels (stained with Laminin) and surrounding glial cells (stained with glial fibrillary acidic protein, GFAP) using immunostaining (**b**, right). Scale bar, 20 μm. (**c**) Visualization of collagen type I (green) in the breast cancer biopsy specimen. Nuclei are counter-stained with propidium iodide (red). Scale bar, 20 μm (**d**) Clearing of human cerebral organoids in sMAX media. Bright-field images of cerebral organoids exhibiting neuromelanins (**d**, left). Images were taken with an EVOS microscope (Thermo Fisher Scientific). Scale bar, 500 μm for whole cerebral organoid images and 50 μm for high-magnified images. 3D whole-mount immunostaining of organoids labeled for Vimentin (**d**, middle). Scale bar, 500 μm. 3D highly magnified image showing cellular arrangements stained with NESTIN (**d**, right). Scale bar, 20 μm. All fluorescent images were acquired using a TCS SP8 confocal laser-scanning microscope and processed with Las X software.

One major requirement for the de-lipidation step is to increase tissue porosity and allow deep antibody penetration^3,4,13,18,38^. Unexpectedly, we found that iMAX significantly quenched CY5- and Alexa-fluorescence signals; thus, we examined the extent of fluorophore quenching using a fluorophotometer to select a suitable fluorophore for MAX imaging (Fig. 5a). Interestingly, loss of the immunofluorescence signal was greater in iMAX-processed specimens than with fluorophore quenching, suggesting that iMAX may interfere with antibody reactions (Supplementary Fig. 5). sMAX may be favored for the 3D imaging of antibody-labeled specimens because sMAX did not exhibit such antibody destabilizing effects. With the sMAX, an increase in the secondary antibody titer and enhancement of laser power could compensate for fluorescence signal quenching (Supplementary Fig. 5). Under these optimized conditions, we were able to image the 3D distribution of signals with many fluorophores in the whole brain (Fig. 5b and Supplementary Movie S5). As mentioned above, because iMAX appears to destabilize the interactions of antibodies with tissue epitopes, inclusion of the fixation step which immobilize the antibody-epitope interaction was required in order to use iMAX as a clearing reagent (Supplementary Fig. 6). In addition to the brain slices, ECM-rich human breast cancer biopsy samples were labeled with antibodies and imaged (Fig. 5c). Finally, human cerebral organoids were cleared and imaged (Fig. 5d). Interestingly, neuromelanins were spontaneously produced by random induction of midbrain-like regions in 3D^39^, and their detailed features were clearly observed in the cleared organoids. Further, detailed neuronal projections and cellular arrangements were detected in the 3D images. Collectively, these results indicated that MAX-based 3D immunolabeling can be applied to a wide variety of biological specimens.

## Discussion

In this study, we propose a one-step reagent, MAX, to process a wide variety of biological specimens using a multistep combination. MAX is a tailored RI adjustment reagent for connective tissue clearing owing to a high RI media with good tissue penetrability. Furthermore, we minimized the caotrophic effect of MXDA and improved tissue penetrability while minimizing specimen deformities. Compared to the benchmarking protocol MACS^28^, MAX does not require serial changes in solutions, making the process simple and rapid. These improvements were mainly achieved by the addition of complementary chemicals, iodixanol and sucrose. Tissue clearing by the MXDA is based on its high RI and hyper-hydrating activity. The hyper-hydrating effect is beneficial in some extent, because it can dilute the light-scattering materials and can promote the diffusion of RI matching media into the tissues^23,33,40^. However, MXDA in high concentration caused dehydration of the tissues owing to the reversal of the tissue osmolarity equilibrium, as we exemplified by the RTT hardening. To achieve diffusion of the MXDA deep into the tissues, this negative feature was obviated by the serial increasing step incubation in the MACS protocol^28^. On the other hand, we identified that the mixture of moderate MXDA concentration with another high-RI reagent such as iodixanol and sucrose can improve the tissue penetrability, allowing the single-step penetration of MXDA into the tissues. Accordingly, inclusion of these chemicals improved the tissue penetrability of the media without significant reduction of the tissue transparency. Furthermore, this also reduced the tissue swelling caused by the MXDA’s hyperhydrating effect. Hyperhydration is associated with the denaturation of the proteins by the caotrophic factors such as urea and MXDA^41^. Thus, we speculate that the kosmotrophic effect of the sucrose/iodixanol counteracted the MXDA-driven tissue swelling.

Considering that the RI matching is one of the most critical steps for the tissue clearing, it is no wonder that there are many single-step methods available^33,40^. However, these RI matching reagents alone are often insufficient for thick tissue imaging, and having difficulty in processing owing to the viscosity and/or rapid dry in the air, and high cost. Our comparison of MAX with other RI matching reagents clearly demonstrated the significant advantage of clearing quality, speed and the cost. For instance, the price for the MAX reagent is > 3 ~ 30 times cheaper than commercially available RIMS (approximately 3 times cheaper in case of iMAX, and 30 times cheaper in case of sMAX). Thus, we propose that MAX is a good alternative for RIMS, especially when large amount of media is required for the large organ clearing and subsequent imaging.

While MAX was designed for connective tissue or ECM-rich organs, it could clear many organs sufficiently. Where MAX alone is insufficient to obtain the desired transparency, other steps such as de-lipidation, de-coloration, and de-calcification can be combined. For instance, the de-lipidation step is necessary for clearing lipid-rich organs such as the liver especially with sMAX media. Although we primarily used the FxClear protocol for de-lipidation, other methods, such as FastClear^42^, PACT^1^, or CUBIC-L^31^ can be alternatively utilized. However, we want to emphasize that the de-lipidation is not necessary in many applications, and the MAX alone can be used for the most imaging procedures as we showed as label-free methods (Fig. 3), DiI/GFP imaging (Fig. 4a–c), and immunofluorescence staining (Fig. 5d). We previously demonstrated that iodixanol, one of the major components of iMAX, has unique features that enhances the contrast of lipid-rich areas^29^ while providing an additional advantage of observation of nerve myelination by label-free imaging. Accordingly, myelination of the iMAX-treated brain during postnatal development could be easily imaged using label-free methods. We discovered that the choice of fluorophore greatly affects the quality of imaging. For instance, nuclear staining dyes such as Hoechst33342 or Syto16, were not compatible with the MAX procedure because of the highly basic pH of MAX (pH > 11), which significantly quenched these fluorescence signals. In contrast, fluorescence signals from propidium iodide, DiI, and many fluorescence-conjugated antibodies are readily detectable alone or in combination with other fluorophores for multi-color imaging.

Collectively, our results propose that MAX can be used for the tissue ECM-rich tissues as a single step reagent, and either alone or in combination of additional steps, it can also utilized for wide variety of the biological specimen with different optical properties.

## Methods

### Biological specimens

#### Rodents

The tendon of the adult Lewis rat was donated by Prof. Park, Jong-Woong (Department of Orthopedic Surgery, Anam Hospital). Tail tendons were obtained from Lewis male rats (12 weeks old) purchased from Orient Bio, Inc. C57BL/6 male mice (P3–8 weeks old) were purchased from Orient Bio Inc. Thy1-YFP transgenic mice were obtained from the Jackson Laboratory. The mice were transcardially perfused with 4% paraformaldehyde (PFA) in 1 × Phosphate-buffered saline (PBS), and organs were isolated and post-fixed in 4% PFA overnight at 4 °C. The back parts containing the vertebrae and surrounding muscles were isolated and processed for de-calcification using a solution containing 14% Ethylenediaminetetraacetic acid (EDTA) in ammonium water (pH = 7.2) for 3 days at 4°C^43^. Next, the specimens were extensively washed with 1 × PBS.

#### Human specimens

Breast mass specimens were obtained by ultrasound (US)-guided biopsy for suspicious breast masses. We used an Aplio 500 US system (Canon Medical Systems, Tokyo, Japan) with a 5–14 MHz linear transducer. The US examinations were performed by a radiologist (B.K.S.) with 21 years of experience in breast imaging. US-guided core needle biopsy was performed using a 14 G needle. The biopsies obtained were placed in formalin bottles for overnight fixation. Human skin was collected from a cadaver for medical student education through an anatomical donation project at Korea University.

#### Adult zebrafish and chicken embryo

Zebrafish were kindly gifted by Prof. Park, Hae Chul (Korea University College of Medicine). Fish were immersed in a 4% PFA solution at room temperature overnight. Pigments in fish skin were bleached with 3% hydrogen peroxide, heated at 37 °C, and incubated for 30 min. For chick embryo, fertilized eggs were obtained from local store (Pulmuone, Korea) and incubated at 37 °C for 12 d. Embryos were isolated from eggs and immersed in 4% PFA at room temperature (25 °C) overnight.

#### Arabidopsis and mushroom

Whole Arabidopsis (5-mm length) were kindly gifted by Prof. Ahn, Ji Hoon (Korea University School of Life Sciences and Biotechonology). They were immersion-fixed with 4% PFA solution in a vacuum chamber at room temperature (25 °C) for 30 min. After removing the fixation solution, the plants were briefly washed with PBS. Subsequently, the chlorophylls were decolored with 15% SDS overnight^5,6^. Mushrooms (*Agaricus bisporus*) were obtained from local grocery stores. After slicing them at 2-mm thickness, they were immersion-fixed overnight. All experiments were performed in accordance with the relevant guidelines and regulations.

#### Human cerebral organoid

Human induced pluripotent stem cell (hiPSC) line UCSD065i-20–3 was purchased from WiCell Research Institute and maintained on Matrigel (Corning, 354277)-coated plates in mTeSR1 (STEMCELL Technologies, 85850). Human cerebral organoids were produced according to a previously described protocol^44^. Briefly, hiPSC colonies were dissociated with Accutase (Innovative Cell Technologies, AT-104), and dissociated cells were seeded onto a 96-well low-attachment plate (9000 cells per well) in low-bFGF hESC medium with ROCK inhibitor. Embryoid bodies (EBs) were formed and cultured for an additional 5–6 days until they grew up to 400 μm in size. The culture conditions were changed to induce primitive neuroepithelia for 4–5 days. When the EBs exhibited a radial arrangement of neuroepithelia, they were embedded in Matrigel droplets and transferred to a neural differentiation medium without vitamin A. After 4 days, the Matrigel-embedded EBs were transferred to an orbital shaker and grown in neural differentiation medium containing vitamin A.

### De-lipidation

De-lipidation was performed using the FxClear method as previously reported^34^. Briefly, fixed tissues were transferred into a cassette and placed in an electrophoretic tissue-clearing (ETC) chamber (X-CLARITY, Logos Biosystems, Korea) containing 2% SDS and 200 mM boric acid in H_2_O (pH 8.5). ETC was processed under the following conditions: 1.2 A, 37 °C for 3 h (tissue slices) or 12 h (whole organs). After clearing, the samples were washed in 0.1 × PBS for 3 h at 25 °C with gentle shaking to remove SDS.

### MAX tissue clearing

Fixed specimens, with or without further processes (de-lipidation and de-coloration), were immersed in the MAX solution overnight in at least five times the volume of the specimen. The MAX solution was composed of iMAX: MXDA (30%) and iodixanol (40%), sMAX: MXDA (30%) and sucrose (44%). To prepare 100 ml of the iMAX solution, 30 ml MXDA solution and 66 ml of iodixanol solution (60%) were mixed overnight at room temperature (25 °C) while stirring. To prepare 100 ml of the sMAX solution, 44 g of sucrose was added to 44 ml of distilled water and stirred with minimal heat. After the powder was completely dissolved, 30 ml of MXDA was added to the solution and stirred thoroughly.

For the comparison, CM and RIMS solutions were used. The CM is composed of sucrose (50%, w/v), urea (25%, w/v) and N,N,N′,N′-tetrakis(2-hydroxypropyl)ethylenediamine (25%, w/v) dissolved in dH_2_O. RIMS was purchased from Logos Biosystems (C13101, Korea).

### Measurements

Tissue transparency (% transparency) was measured using a previously described protocol^26^. Briefly, images of RTT or 1-mm thick organ slices were obtained using a transillumination microscopic system (EVOS M5000, Thermo Fisher Scientific, Waltham, MA, USA) with fixed light intensity. The size and transparency of the specimens were measured from the images using ImageJ (NIH, USA). The grey value of the cleared sample image was used to measure tissue transparency, which was normalized to the image background.

### Vasculature labeling with CM-DiI

Adult C57BL/6 mice were anesthetized with urethane and transcardially perfused with saline containing 0.01% CM-DiI solution for vasculature labeling^45^, followed by fixation with 4% paraformaldehyde. CM-DiI (Thermo Fisher Scientific) is an aldehyde-fixably modified version of the DiI. The fixed sample was cut to a thickness of 1 mm and incubated with the iMAX solution overnight for 3D imaging.

### Immunostaining

After de-lipidation process, tissue specimens were incubated with primary antibodies in 6% (w/v) bovine serum albumin (BSA), 0.2% (v/v) Triton X-100, and 0.01% (w/v) sodium azide in 0.1 × PBS for 1–2 d in a 37 °C shaker. The antibodies used in this study were as follows MBP (1:000, Ab40390, Abcam, RRID:AB_1141521), Calbindin D-28K (1:500, 300, Swant, RRID:AB_10000347) TH (1:1000, AB152, Millipore, RRID:AB_390204), laminin (1:1000, L9393, Sigma, RRID:AB_477163), GFAP (1:500, 13-0300, Invitrogen, RRID:AB_2532994), and collagen type 1 (1:4500, ab34710, Abcam, RRID:AB_731684), Parvalbumin (1:1000, 195004, Synaptic Systems, RRID:AB_2156476), NeuN (1:1000, ABN78, Millipore, RRID:AB_10807945). The samples were then washed with 0.1 × PBS at least twice and incubated with appropriate secondary antibodies (1:100) for 1–2 days in a 37 °C shaker. In some cases, the nuclei were counter-stained with propidium iodide (1:1000, P3566, Thermo Fisher Scientific), and the samples were incubated with MAX solution overnight for 3D imaging.

Whole cerebral organoids were fixed with 4% PFA for 1 h, washed several times with PBST (0.1% Triton X-100 in 1 × PBS), and incubated overnight with blocking solution (6% BSA, 0.2% Triton X-100, and 0.01% sodium azide in PBS). The cerebral organoids were then incubated for 3 days with primary antibodies against vimentin (1:500, ab1620, Millipore, RRID:AB_90774) and nestin (1:400, MAB1259, R&D, RRID:AB_2251304). The samples were then washed and incubated with the appropriate secondary antibodies for 3 days. Subsequently, the samples were washed with PBST and incubated with the MAX solution overnight for optical clearing.

### 3D imaging

Fluorescence images were acquired using a TCS SP8 confocal laser-scanning microscope (Leica, Germany) in the serial z-scanning mode. The acquired z-series images were processed using the LAS X software version 3.5.5.19976 (Leica, Wetzlar, Germany) and IMARIS software version 9.0 (Bitplane, Belfast, United Kingdom)^46,47^. The SPIM imaging system (Logos biosystems, Anyang, South Korea) has also been used for label-free imaging of mouse brain slices^48^. A Z-stack of a mouse brain was imaged using an excitation laser of 638 nm without the emission filter. The thickness of the light sheet was 2 μm, and 4 × objective lens (NA0.13) was used for image acquisition., volume rendering of Z‐stack acquisition was obtained using Amira software version 5.3.3 (Thermo Fisher Scientific, USA) and IMARIS software version 9.0 (Bitplane, Belfast, United Kingdom)^9,46^.

### Fluorescence measurements

An EnSpire Multimode Microplate Reader (PerkinElmer, Waltham, MA, USA) was used to detect the fluorescence intensity in the MAX solution. The secondary antibodies conjugated to different fluorophores were diluted 10 times with PBS, iMAX, and sMAX in a 96-well black microplate. After incubation at room temperature (25 °C) for 18 h, relative fluorescence units (RFUs) were acquired. The RFU values of the MAX solution were normalized to those of PBS. Fluorescence intensity in the cortical area of the captured images from the immunolabeled brain slices was measured using ImageJ, as previously reported^49^.

### Statistical analyses

Statistical analysis was performed using the GraphPad Prism 9 software version 9.3.1 (GraphPad, CA, USA). All data are expressed as the mean ± SEM, with sample sizes of n ≥ 3, unless stated otherwise. The analysis methods used included ordinary one-way ANOVA followed by Tukey’s post hoc test, and two-way ANOVA with Bonferroni correction, as appropriate. The control condition was used to determine statistically significant differences for multiple comparisons, and an unpaired two-tailed t-test was used to compare the two groups.

### Ethics declarations

All procedures in the present study were conducted in accordance with the ethical standards of the institutional research committee. All animal handling and husbandry procedures were approved by the Korea University Institutional Animal Care and Use Committee (KOREA-2019-0014 and KOREA-2021-0106). All procedures for human breast tissue were approved by the Institutional Review Board of Korea University Ansan Hospital (IRB no. 2020AS0113), and written informed consent was obtained from all participants. The procedures for human skin were approved by the Institutional Review Board of the Korea University College of Medicine. The authors declare that the study is carried out in accordance with the ARRIVE guidelines (https://arriveguidelines.org).

## Supplementary Information

Supplementary Figures.

Supplementary Video 1.

Supplementary Video 2.

Supplementary Video 3.

Supplementary Video 4.

Supplementary Video 5.

## Data Availability

The datasets generated during and/or analyzed during the current study are available from the corresponding author on reasonable request.

## Abbreviations

3D: 3-Dimensional
BSA: Bovine serum albumin
EBs: Embryoid bodies
ECM: Extracellular matrix
EDTA: Ethylenediaminetetraacetic acid
ETC: Electrophoretic tissue-clearing
GFP: Green fluorescence protein
hiPSCs: Human induced pluripotent stem cells
MAX: MXDA-based aqueous RI adjustment solution X
MXDA: Meta-xylenediamine
PBS: Phosphate-buffered saline
PFA: Paraformaldehyde
RFUs: Relative fluorescence units
RI: Refractive index
RTT: Rat tail tendon
SDS: Sodium dodecyl sulfate
SPIM: Selective-plane ilumination microscopy

## References

[1] Yang B (2014). Single-cell phenotyping within transparent intact tissue through whole-body clearing. Cell.

[2] Chung K (2013). Structural and molecular interrogation of intact biological systems. Nature.

[3] Susaki EA (2014). Whole-brain imaging with single-cell resolution using chemical cocktails and computational analysis. Cell.

[4] Ueda HR (2020). Tissue clearing and its applications in neuroscience. Nat. Rev. Neurosci..

[5] Kurihara D, Mizuta Y, Sato Y, Higashiyama T (2015). ClearSee: A rapid optical clearing reagent for whole-plant fluorescence imaging. Development.

[6] Tofanelli R, Vijayan A, Scholz S, Schneitz K (2019). Protocol for rapid clearing and staining of fixed Arabidopsis ovules for improved imaging by confocal laser scanning microscopy. Plant Methods.

[7] Konno A, Okazaki S (2018). Aqueous-based tissue clearing in crustaceans. Zool. Lett..

[8] Pende, M. *et al.* A versatile depigmentation, clearing, and labeling method for exploring nervous system diversity. *Sci. Adv.* **6**, eaba0365 (2020). 10.1126/sciadv.aba0365 PMC7259959 32523996

[9] Jährling N, Becker K, Schönbauer C, Schnorrer F, Dodt H-U (2010). Three-dimensional reconstruction and segmentation of intact Drosophila by ultramicroscopy. Front. Syst. Neurosci..

[10] Pende M (2018). High-resolution ultramicroscopy of the developing and adult nervous system in optically cleared Drosophila melanogaster. Nat. Commun..

[11] Jing D (2018). Tissue clearing of both hard and soft tissue organs with the PEGASOS method. Cell Res..

[12] Pan C (2016). Shrinkage-mediated imaging of entire organs and organisms using uDISCO. Nat. Methods.

[13] Lee E (2016). ACT-PRESTO: Rapid and consistent tissue clearing and labeling method for 3-dimensional (3D) imaging. Sci. Rep..

[14] Belle, M. *et al.* Tridimensional visualization and analysis of early human development. *Cell* **169**, 161–173.e112 (2017). 10.1016/j.cell.2017.03.008 28340341

[15] Zhao, S. *et al.* Cellular and molecular probing of intact human organs. *Cell* **180**, 796-812.e719 (2020). 10.1016/j.cell.2020.01.030 PMC7557154 32059778

[16] Costantini I, Cicchi R, Silvestri L, Vanzi F, Pavone FS (2019). In-vivo and ex-vivo optical clearing methods for biological tissues. Biomed. Opt. Express.

[17] Tainaka K, Kuno A, Kubota SI, Murakami T, Ueda HR (2016). Chemical principles in tissue clearing and staining protocols for whole-body cell profiling. Annu. Rev. Cell Dev. Biol..

[18] Richardson DS, Lichtman JW (2015). Clarifying tissue clearing. Cell.

[19] Ke M-T, Fujimoto S, Imai T (2013). SeeDB: a simple and morphology-preserving optical clearing agent for neuronal circuit reconstruction. Nat. Neurosci..

[20] Kuwajima T (2013). ClearT: a detergent-and solvent-free clearing method for neuronal and non-neuronal tissue. Development.

[21] Renier N (2014). iDISCO: A simple, rapid method to immunolabel large tissue samples for volume imaging. Cell.

[22] Tainaka K (2014). Whole-body imaging with single-cell resolution by tissue decolorization. Cell.

[23] Kim JY (2018). BrainFilm, a novel technique for physical compression of 3D brain slices for efficient image acquisition and post-processing. Sci. Rep..

[24] Lee B (2021). Sensitive label-free imaging of brain samples using FxClear-based tissue clearing technique. Iscience.

[25] Lee E, Kim HJ, Sun W (2016). See-through technology for biological tissue: 3-dimensional visualization of macromolecules. Int. Neurourol. J..

[26] Kim JH (2018). Optimizing tissue-clearing conditions based on analysis of the critical factors affecting tissue-clearing procedures. Sci. Rep..

[27] Lee E (2019). High-performance acellular tissue scaffold combined with hydrogel polymers for regenerative medicine. ACS Biomater. Sci. Eng..

[28] Zhu J (2020). MACS: rapid aqueous clearing system for 3D mapping of intact organs. Adv. Sci..

[29] Boothe T (2017). A tunable refractive index matching medium for live imaging cells, tissues and model organisms. Elife.

[30] Na M (2022). Sodium cholate-based active delipidation for rapid and efficient clearing and immunostaining of deep biological samples. Small Methods.

[31] Tainaka, K. *et al.* Chemical landscape for tissue clearing based on hydrophilic reagents. *Cell Rep.* **24**, 2196–2210. e2199 (2018). 10.1016/j.celrep.2018.07.056 30134179

[32] Zhu J (2021). Tissue optical clearing for 3D visualization of vascular networks: A review. Vascul. Pharmacol..

[33] Yu T, Zhu J, Li D, Zhu D (2021). Physical and chemical mechanisms of tissue optical clearing. Iscience.

[34] Choi J, Lee E, Kim JH, Sun W (2019). FxClear, a free-hydrogel electrophoretic tissue clearing method for rapid de-lipidation of tissues with high preservation of immunoreactivity. Exp. Neurobiol..

[35] Lyon, H. *Theory and Strategy in Histochemistry: a Guide to the Selection and Understanding of Techniques*. (Springer, 1991).

[36] Hedhly, A., Vogler, H., Eichenberger, C. & Grossniklaus, U. Whole-mount clearing and staining of Arabidopsis flower organs and siliques. *JoVE (Journal of Visualized Experiments)*, e56441 (2018). 10.3791/56441 PMC5933498 29708535

[37] Quarles RH, Macklin WB, Morell P (2006). Myelin formation, structure and biochemistry. Basic Neurochem. Mol. Cell. Med. Asp..

[38] Murray E (2015). Simple, scalable proteomic imaging for high-dimensional profiling of intact systems. Cell.

[39] Jo J (2016). Midbrain-like organoids from human pluripotent stem cells contain functional dopaminergic and neuromelanin-producing neurons. Cell Stem Cell.

[40] Zhu D, Larin KV, Luo Q, Tuchin VV (2013). Recent progress in tissue optical clearing. Laser Photon. Rev..

[41] Su Z, Dias CL (2017). Molecular interactions accounting for protein denaturation by urea. J. Mol. Liq..

[42] Perbellini F (2017). Free-of-Acrylamide SDS-based Tissue Clearing (FASTClear) for three dimensional visualization of myocardial tissue. Sci. Rep..

[43] Jin R (2013). Activation of NF-kappa B signaling promotes growth of prostate cancer cells in bone. PLoS ONE.

[44] Lancaster MA (2013). Cerebral organoids model human brain development and microcephaly. Nature.

[45] Li Y (2008). Direct labeling and visualization of blood vessels with lipophilic carbocyanine dye DiI. Nat. Protoc..

[46] Li A (2010). Micro-optical sectioning tomography to obtain a high-resolution atlas of the mouse brain. Science.

[47] Chaudhry A, Shi R, Luciani DS (2020). A pipeline for multidimensional confocal analysis of mitochondrial morphology, function, and dynamics in pancreatic β-cells. Am. J. Physiol. Endocrinol. Metab..

[48] Huisken J, Stainier DY (2007). Even fluorescence excitation by multidirectional selective plane illumination microscopy (mSPIM). Opt. Lett..

[49] Jensen EC (2013). Quantitative analysis of histological staining and fluorescence using ImageJ. Anat. Rec..

