# Supplementary material for: MAX: a simple, affordable, and rapid tissue clearing reagent for 3D imaging of wide variety of biological specimens
Source: Sci Rep. 2022 Nov 14;12:19508. doi: 10.1038/s41598-022-23376-6 (PMC9663452; doi:10.1038/s41598-022-23376-6)
Supplement: Supplementary file 1 — Supplementary Figures. [file 41598_2022_23376_MOESM1_ESM.docx]

Supplementary Information

**MAX: a Simple, Affordable, and Rapid Tissue Clearing Reagent for 3D Imaging of Wide Variety of Biological Specimens**

Boram Lee, Ju-Hyun Lee, Dai Hyun Kim, Eun Sil Kim, Bo Kyoung Seo, Im Joo Rhyu, **Woong Sun***

B. Lee, J.H. Lee, D.A. Kim, I.J. Rhyu, W.Sun

Department of Anatomy, Brain Korea 21 Plus Program for Biomedical Science, Korea University College of Medicine, Seoul 02841, Republic of Korea

**E-mail: woongsun@korea.ac.kr**

D.H. Kim

Department of Dermatology, Korea University College of Medicine, Seoul 02841, Republic of Korea

E.S. Kim, B.K. Seo

Department of Radiology, Korea University Ansan Hospital, Korea University College of Medicine, 123 Jeokgeum-ro, Danwon-gu, Ansan city, Gyeonggi-do 15355, Korea


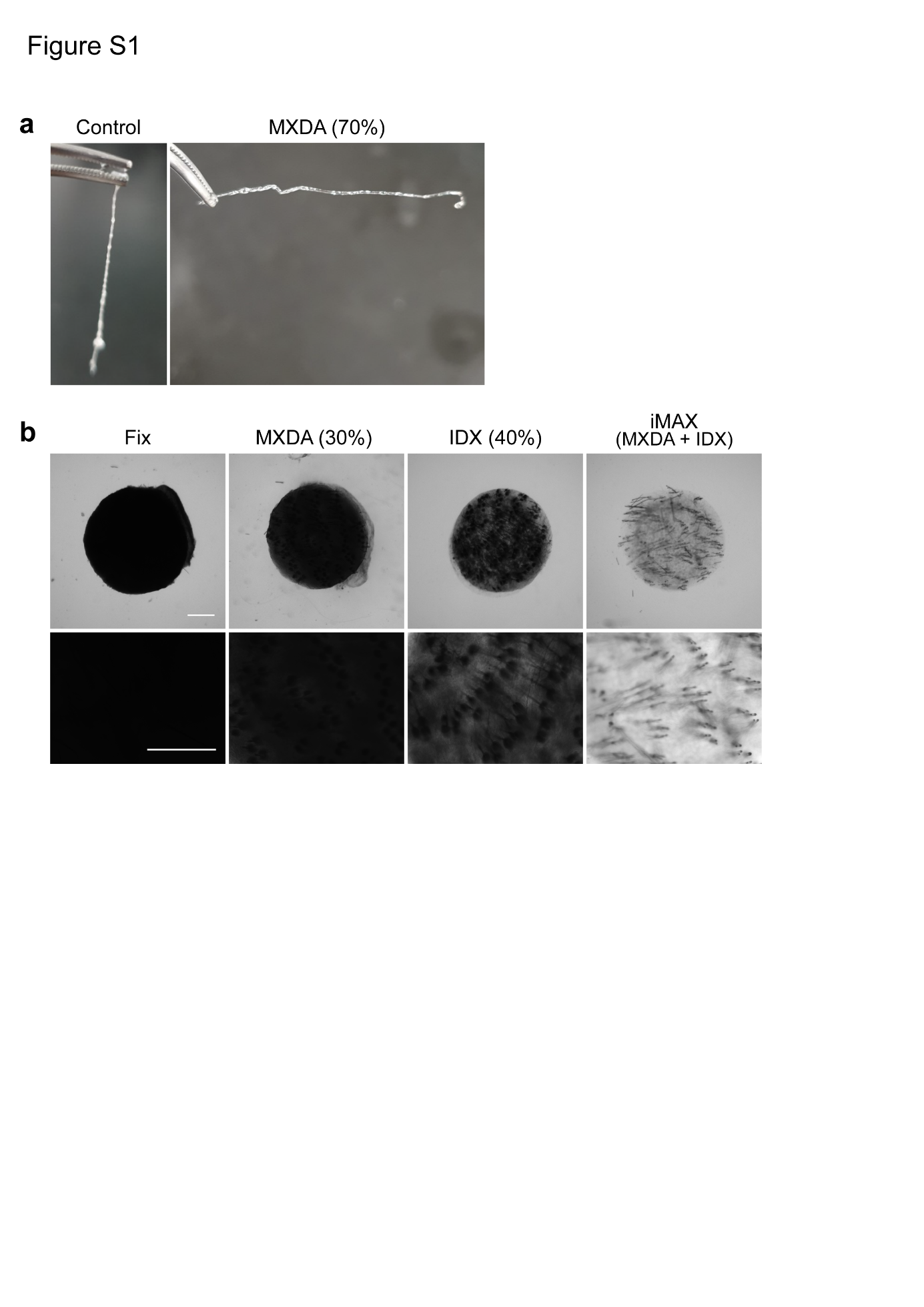


**Figure S1.** (a) Hardening of the rat tail tendon (RTT) after 70% MXDA incubation. Note that MDXA-treated RTT became hard and could be lifted by forceps. (b) Transparency of the mouse skin punches in MXDA, iodixanol (IDX) and iMAX solutions. Large magnification images are presented at the bottom of the panel. Note that hair in the skin sample were not cleared. Scale bars, 500 μm.

**
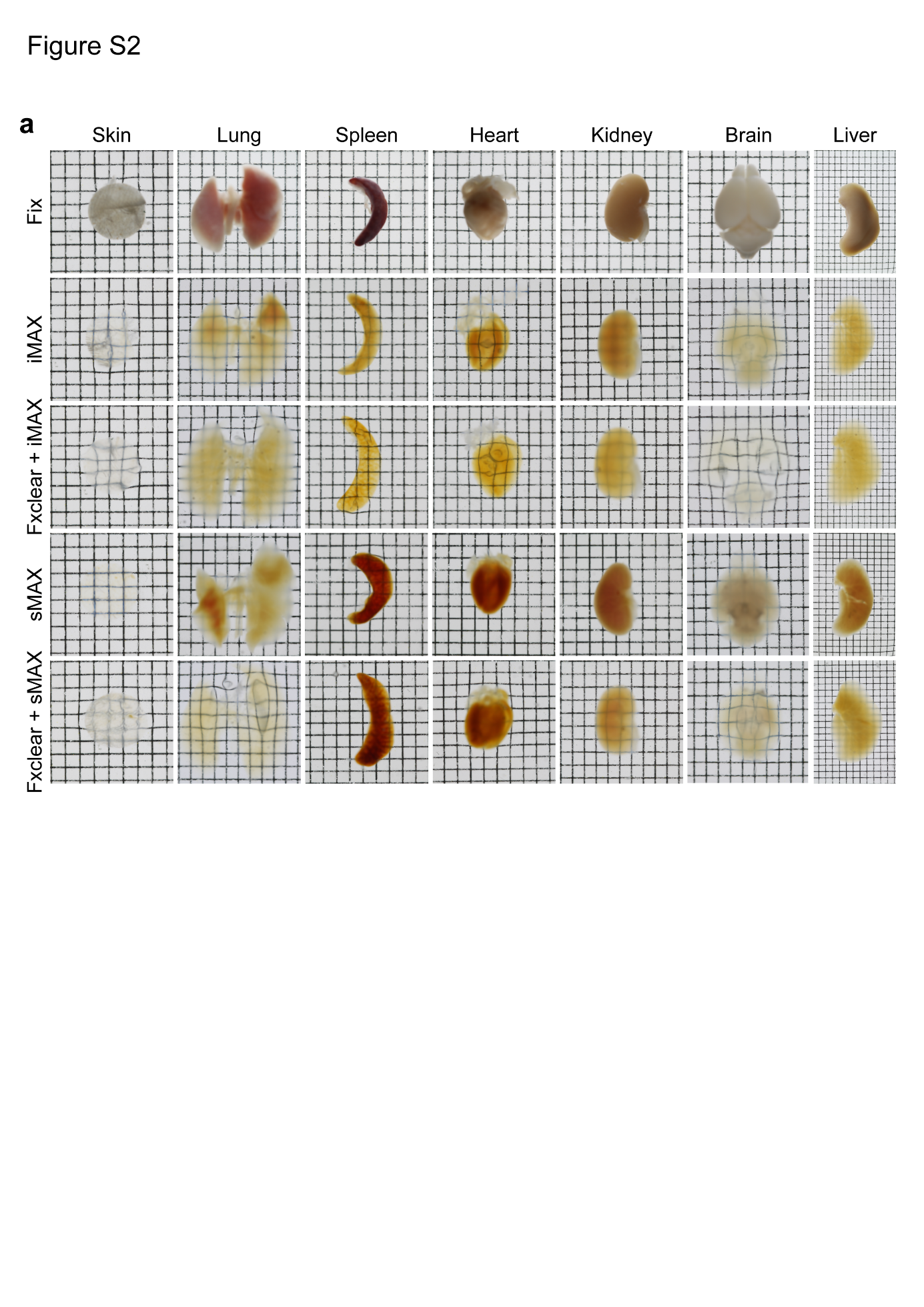
**

**Figure S2.** Full set of organ-clearing results in MAX solutions. Fixed mouse organs (skin, lung, spleen, heart, kidney, brain, and liver) are immersed in the iMAX or sMAX solutions overnight with or without FxClear de-lipidation steps. Note that the images of brain and livers are duplicated with main Figure 2a. All specimens are placed on the 2-mm grid paper.


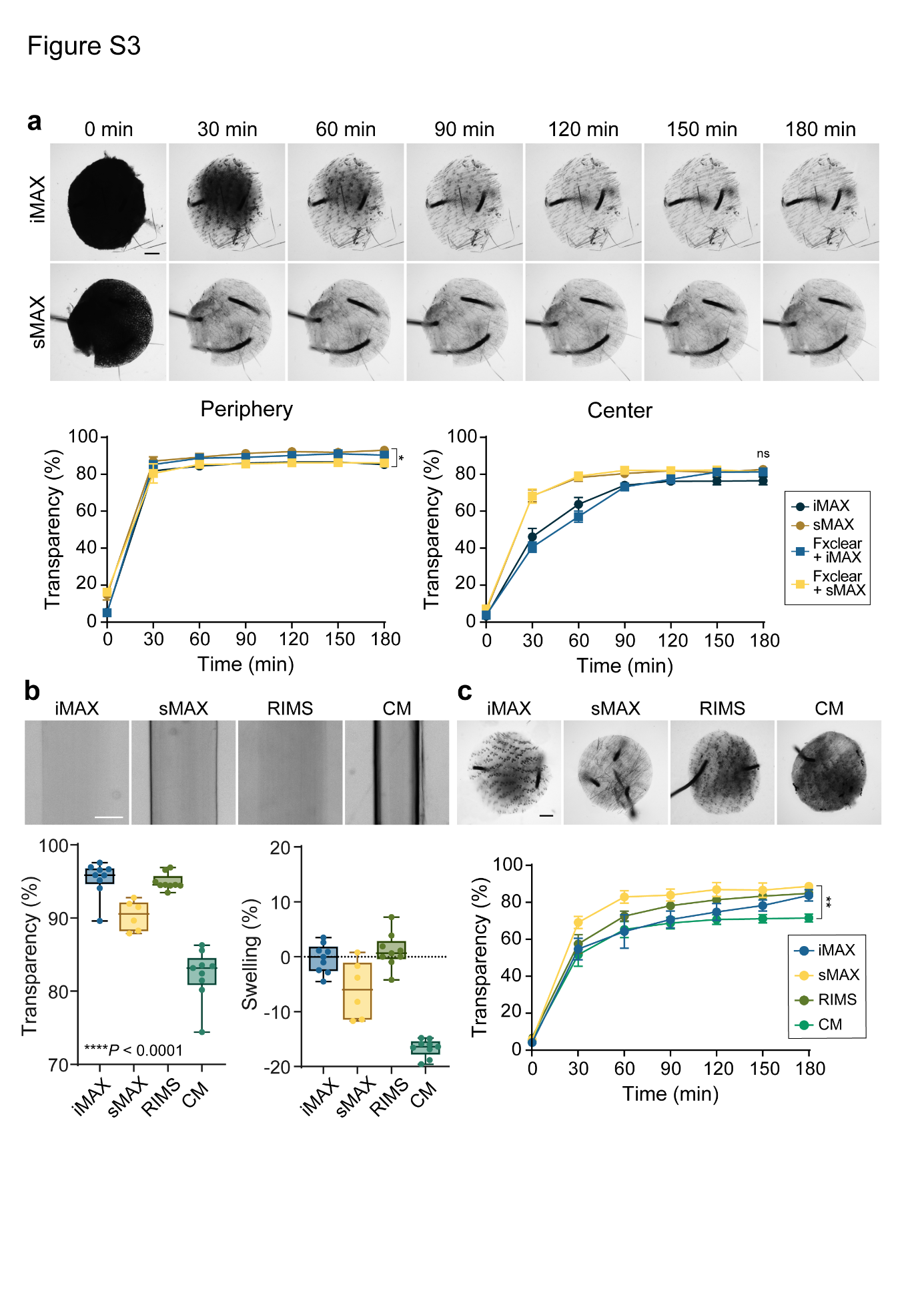


**Figure S3.** (a) Time-course changes of the skin specimen clearing in the iMAX or sMAX solutions. Black lines in the time series images are tungsten needles immobilizing the specimen during the experiments. Scale bar, 500 μm. Low panels are the quantification results from specimen periphery (left) and center (right) regions. N=4 animals. (b) Comparisons of the RTT clearing among the MAX reagents with other single-step clearing reagents, RIMS and CUBIC-mount (CM). Low panels are the quantification results of %transparency (left) and %swelling (right). Standard one-way ANOVA with Tukey’s multiple comparison test was used for multiple comparisons. N= 3 animals. Data spots were obtained from 1-3 different RTTs from individual animals. Scale bar, 100 μm. (c) Time-course changes of skin specimen clearing in different RI matching media. Upper images were captured at 30-min after the immersion. Low panel shows the quantification result of the tissue clearing. N=4. Scale bar, 500 μm. The unpaired two-tailed t-test was used to analyze the differences in maximum transparency (180 min) between the groups (ns, not significant; *p < .05; **p < .01; panel a and c).


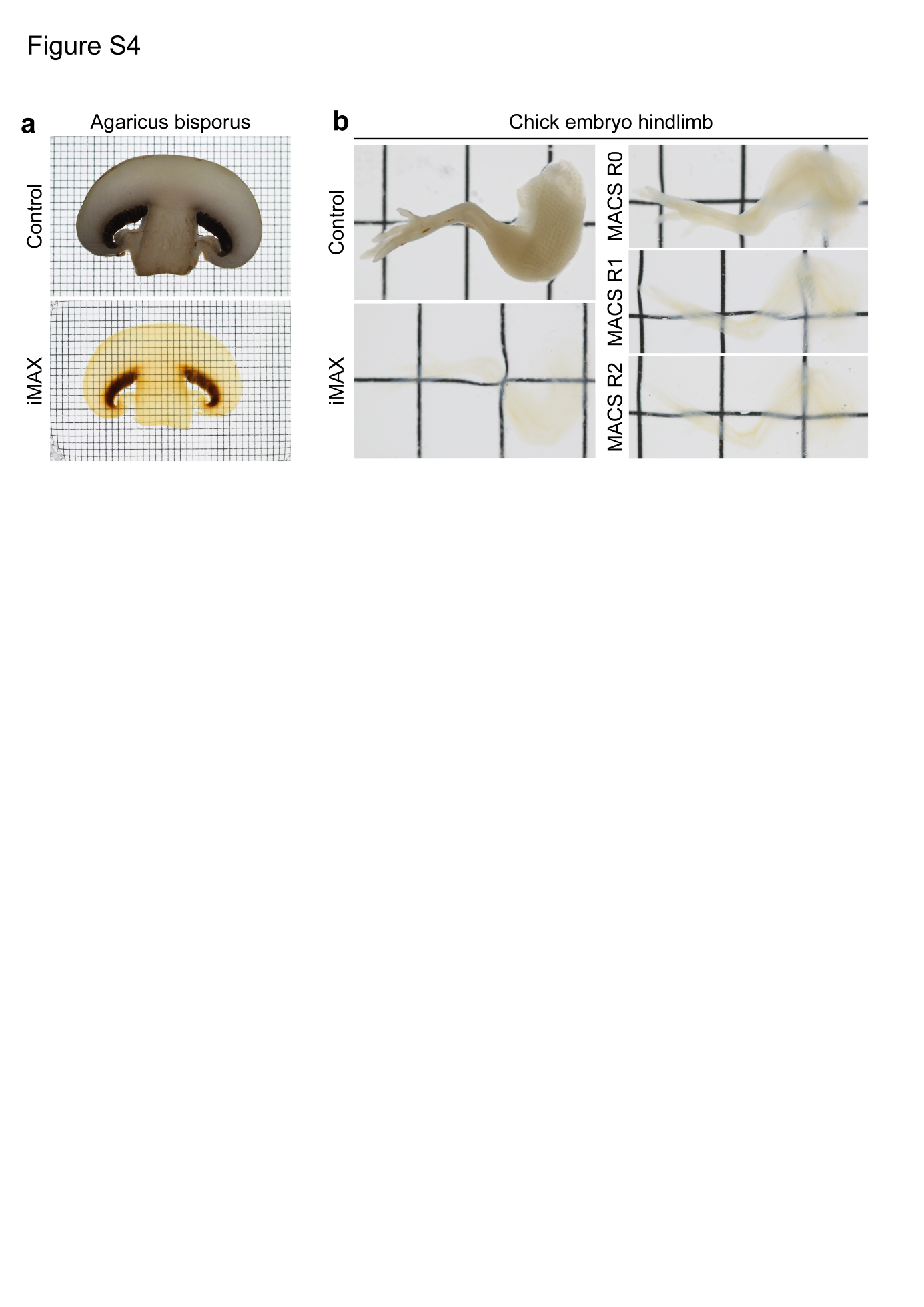


**Figure S4.** (a) Mushroom (*Agaricus bisporus*) was cleared in iMAX solution. (b) Comparison of iMAX and MACS procedure. Legs of chick embryo (E12) are immersed in iMAX overnight (left) or the steps of MACS procedure (MACS R0-R2) is executed for total of 3 days by overnight incubation at each step. All specimens are placed on the 2-mm (a, b) or 7-mm (c) grid paper.


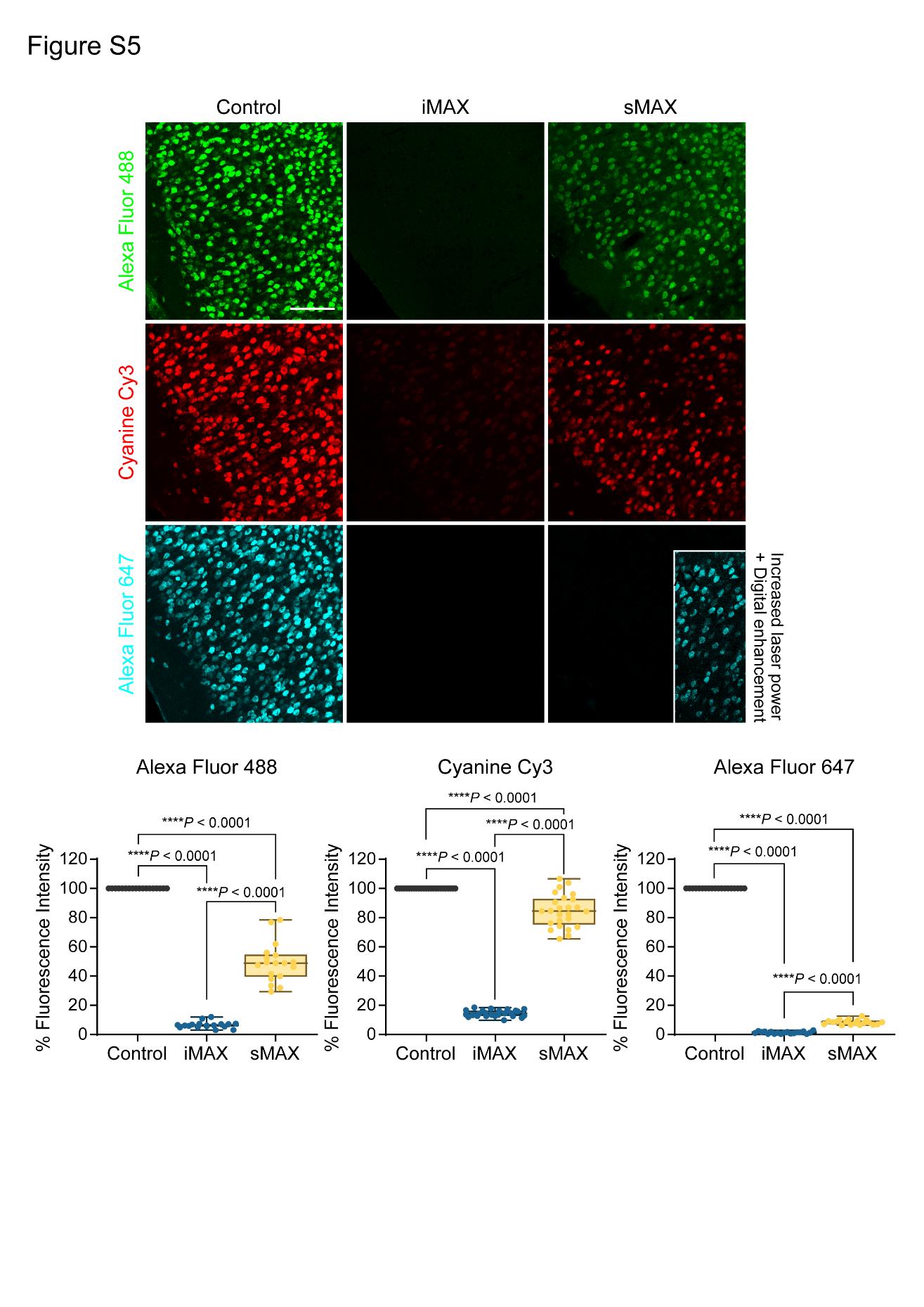


**Figure S5.** Immunofluorescence staining intensities in the MAX solutions. NeuN-labeled brain slices were incubated with different fluorophore-tagged secondary antibodies and immersed in iMAX or sMAX solution. Image in the inset is the result of signal enhancement through post-processing after capturing of signal with 5-fold higher laser power. Lower graphs show the quantification of the fluorescence intensities from different fluorophores. Unpaired student’s t-test was used for comparing two groups. Scale bar, 100 μm


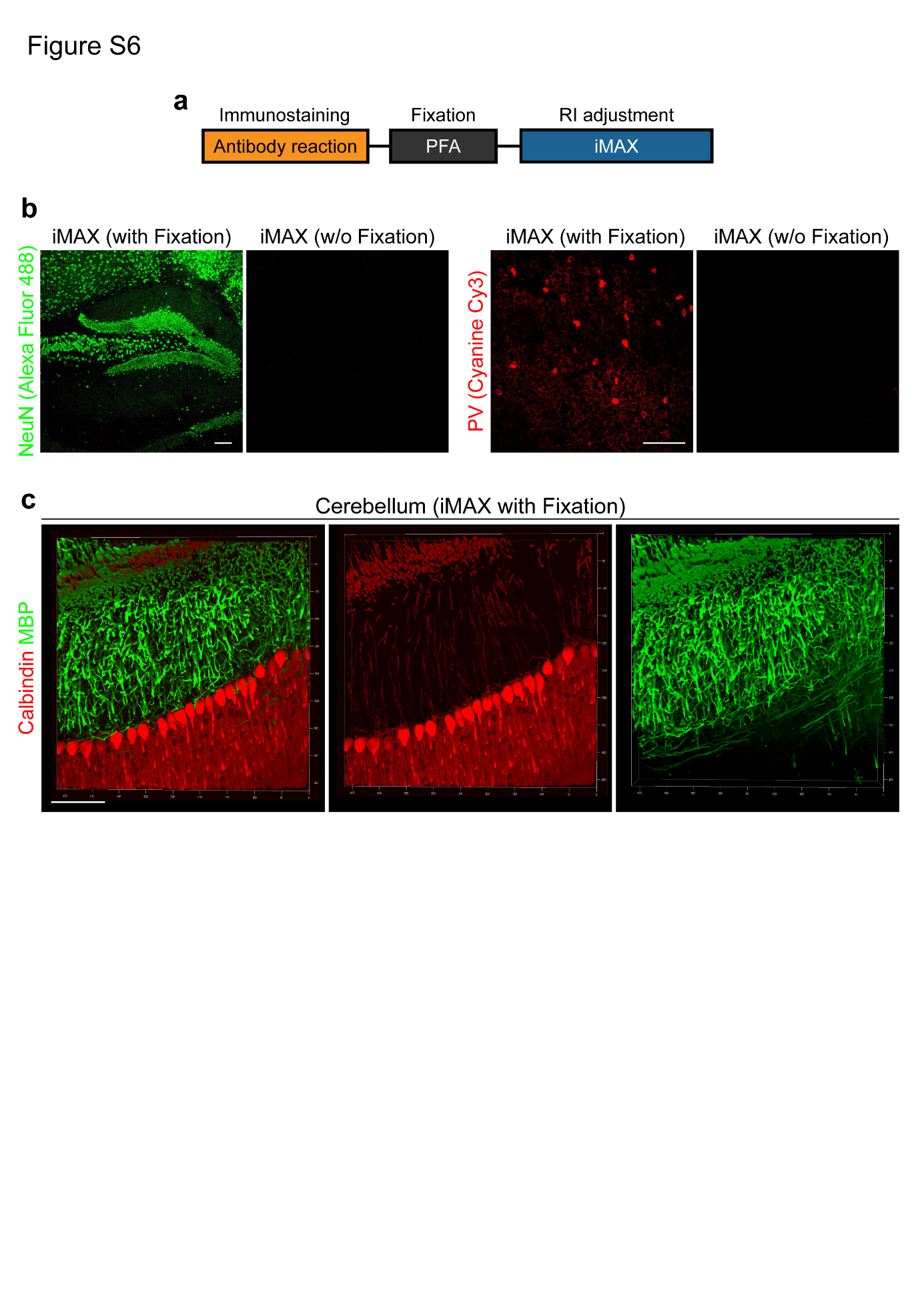


**Figure S6.** 3D Immunofluorescence imaging with iMAX solution. (a) The fixation step before iMAX is added to stabilize antibody binding to epitopes. (b) Comparison of the immunostaining results with or without post-fixation step. Bran slices were stained with NeuN (Alexa Flour 488) or Parvalbumin (PV, Cy3). (c) Purkinje cells in the mouse cerebellum tissue were labeled by Calbindin (Cyanine3), and the myelinated fibers were stained with myelin basic protein (MBP, Alexa Fluor 488). Scale bar, 100 μm

Movie S1. Time-lapse imaging of mouse skin, brain, and kidney slices in the iMAX solution.

Movie S2. Label-free, 3D visualization of adult mouse brain imaged by SPIM.

Movie S3. 3D imaging of DiI-labeled vasculatures in the kidney glomeruli.

Movie S4. 3D imaging of DiI-labeled vascularture in the whole Thy1-GFP mouse brain

Movie S5. 3D immunofluorescence imaging of astrocytes (GFAP, green) and blood vessels (Laminin, magenta) in the mouse brain.
